# Effect of elbow joint angles on electromyographic activity versus force relationships of synergistic muscles of the triceps brachii

**DOI:** 10.1371/journal.pone.0252644

**Published:** 2021-06-03

**Authors:** Hiroshi Akima, Hisashi Maeda, Teruhiko Koike, Koji Ishida

**Affiliations:** 1 Research Center of Health, Physical Fitness & Sports, Nagoya University, Nagoya, Aichi, Japan; 2 Graduate School of Education and Human Development, Nagoya University, Nagoya, Aichi, Japan; 3 Graduate School of Medicine, Nagoya University, Nagoya, Aichi, Japan; University of Pittsburgh, UNITED STATES

## Abstract

The electromyographic (EMG) activity and force relationship, i.e. EMG-force relationship, is a valuable indicator of the degree of the neuromuscular activation during isometric force production. However, there is minimal information available regarding the EMG-force relationship of individual triceps brachii (TB) muscles at different elbow joint angles. This study aimed to compare the EMG-force relationships of the medial (TB-Med), lateral (TB-Lat), and long heads (TB-Long) of the TB. 7 men and 10 women performed force matching isometric tasks at 20%, 40%, 60%, and 80%maximum voluntary contraction (MVC) at 60°, 90°, and 120° of extension. During the submaximal force matching tasks, the surface EMG signals of the TB-Med, TB-Lat, and TB-Long were recorded and calculated the root mean square (RMS). RMS of each force level were then normalized by RMS at 100%MVC. For the TB-Med, ultrasonography was used to determine the superficial region of the muscle that faced the skin surface to minimize cross-talk. The joint angle was monitored using an electrogoniometer. The elbow extension force, elbow joint angle, and surface EMG signals were simultaneously sampled at 2 kHz and stored on a personal computer. The RMS did not significantly differ between the three muscles, except between the TB-Med and TB-Lat during 20%MVC at 60°. The RMS during force levels of ≥ 60%MVC at 120° was significantly lower than that at 60° or 90° for each muscle. The sum of difference, which represents the difference in RMS from the identical line, did not significantly differ in any of the assessed muscles in the present study. This suggests that a relatively smaller neuromuscular activation could be required when the elbow joint angle was extended. However, neuromuscular activation levels and relative force levels were matched in all three TB synergists when the elbow joint angle was at 90° or a more flexed position.

## Introduction

The force exerted by the muscle depends on the motor unit (MU) activation patterns and the mechanical properties of the muscle fibers and muscle-tendon complex. It is also well known that changes in the joint angle or muscle length have a great effect on maximal or submaximal muscle force [1]. Thus, the change in the muscle length has a great impact on the muscle force output. Previous studies have used the relationship between the root mean square (RMS) of surface electromyographic (EMG) signal and the generated force, i.e. RMS-force relationship, as a valuable indicator to estimate the recruitment and/or firing rate of MUs with changes in muscle length and exerted force levels in skeletal muscle [2, 3].

Previous studies have investigated the RMS-force relationship in the knee extensors [4–7], elbow extensors and flexors [8–10], deltoid muscle [11], and finger muscles [11]. The RMS-force relationship is affected by the anatomical structure, degree of synergistic action of other muscle groups, co-contraction among antagonist muscle groups [2, 3], and/or motoneuron stimulation rate [12]. Furthermore, the RMS-force relationship differs even within the synergistic muscles of a given muscle group, such as the triceps brachii (TB) [13] and quadriceps femoris [14]. Harwood et al. [13] showed that recruitment thresholds of motor units (MUs) of the long head of the TB (TB-Long) were significantly higher than that of the lateral head (TB-Lat). This suggests that the TB-Long and TB-Lat have different MUs recruitment properties with changes in muscle force. Furthermore, in the knee joint, the normalized RMS-force relationship of the quadriceps femoris was shifted down by extension of joint angles [14], implying changes in joint angle affects EMG activation patterns. However, it is not clear whether the normalized RMS-force relationship significantly differs among the three synergistic TB muscles with changes in the elbow joint angle. Accumulating data on the neuromuscular activity of the TB muscle with changes in elbow joint angle will be important for establishing accurate neuromusculoskeletal modelling for estimating muscle force from EMG in the future.

The EMG activity of the quadriceps femoris is affected by anatomical characteristics such as muscle length [1, 14, 15]; the normalized RMS-force relationship shifts downward with knee extension, and the deeper vastus intermedius muscle is the most affected of the synergistic muscles [14]. This downward shift of the normalized EMG-force relationship in the vastus intermedius may be related to the change in the muscle length, which has been shown to induce a change in the MU recruitment threshold [16] and/or MU discharge rate [17, 18]. In addition, the EMG activity of the vastus intermedius decreases with knee extension in assessments performed using needle EMG [1, 14, 15] and surface EMG [1, 14, 15]. However, the joint angle reportedly has no significant effect on the normalized EMG-force relationship of the biceps brachii and brachioradialis muscles [8]. In contrast, although the normalized EMG-force relationship of the TB muscles is not affected by joint angles from 60° to 135° (180° = full extension), it is significantly affected by joint angles from 150° to 170° [8].

The TB is the largest muscle around the elbow joint [19, 20] and is composed of the medial (TB-Med), TB-Lat, and TB-Long heads, which extend the elbow. The TB-Med is located at the deeper region, the fleshy origin is along the posterior humerus between the insertion of the teres major muscle and the olecranon fossa. The TB-Lat and TB-Long are located superficially, originating from the posterior surface of the superior humerus and infraglenoid tuberosity of the scapula, respectively, with two tendons at the proximal and distal ends [21, 22]. All three heads of the TB insert by a common, broad tendon into the posterior surface of the olecranon and into the deep antebrachial fascia on each side of it [22]. These differences in the anatomical properties of superficial versus deeply located muscles may affect the normalized EMG-force relationship of the TB muscle group, as shown in the quadriceps femoris [14]. Furthermore, neurophysiological characteristics greatly influence the MU recruitment and rate coding characteristics [13, 17], which are closely related to the linearity or non-linearity of the normalized EMG-force relationship. For example, Harwood et al. [13] demonstrated that the MU recruitment threshold does not significantly differ between the TB-Lat and TB-Long during an isometric elbow extension task. This suggests that MUs of similar sizes will be recruited with increasing force. However, almost all previous studies have focused on the TB-Lat, while the functional characteristics of the TB-Med and TB-Long have not been well clarified [8, 17, 23].

The present study aimed to assess the normalized EMG-force relationship of the TB-Med, TB-Lat, and TB-Long during isometric elbow extensions of 60°, 90°, and 120°. We hypothesized that the normalized EMG-force relationship of the deeper TB-Med shifts downward with elbow extension, i.e. shorter muscle length [14]. Unlike the TB-Lat and TB-long, the TB-Med attaches to the humerus over a wide area without going through tendon tissue. Therefore, the normalized EMG-force relationship of the TB-Med, but not TB-long and TB-Lat, will be directly and sensitively affected by changes in the elbow joint angle and muscle forces.

## Materials and methods

### Subjects

The present study included seven men (age, 20.7 ± 0.4 years; height, 171.3 ± 2.7 cm; weight, 63.1 ± 3.8 kg) and 10 women (age, 19.6 ± 0.5 years; height, 160.0 ± 1.6 cm; weight, 52.2 ± 0.9 kg). The study purpose, procedures, risks and benefits were explained verbally and in written form, and written informed consent was obtained from each subject before study commencement. The experiment protocols were approved by the human research ethics committee of the Graduate School of Medicine, Nagoya University (2017–0321), and were in accordance with the Declaration of Helsinki.

### Experimental protocol

One week prior to the experiment, all subjects performed a few practice repetitions of the MVC force testing using their right arm at any two joint angles, and performed the force matching task. On the experiment day, subjects performed the MVC force test with a sustained force phase of about 3 seconds, and a force matching task with a steady state phase of exerted force for 5 seconds using their right arm at 20%, 40%, 60%, and 80%MVC at angles of 60°, 90°, and 120° in random order. During the task, the surface EMG signals of the TB-Med, TB-Lat, and TB-Long muscles were synchronously recorded along with the elbow extension force and joint angle.

### Maximal and submaximal isometric elbow extension force task

**Fig 1:**
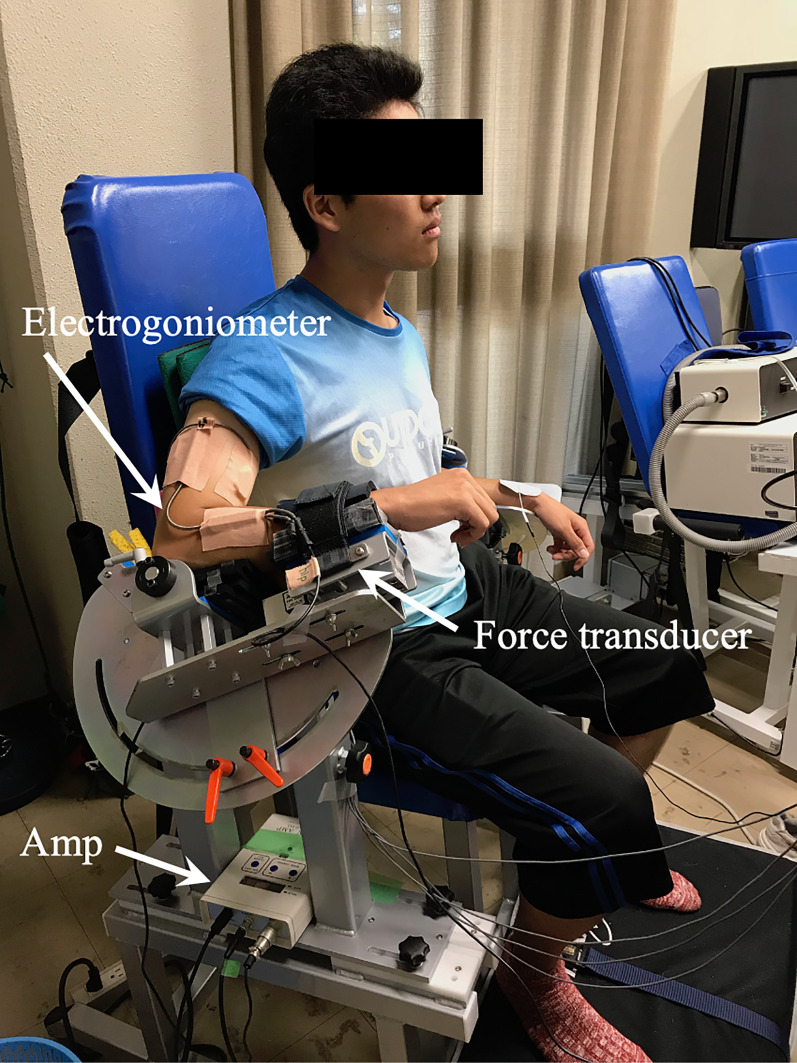
Experimental setup of elbow extension tasks and placement of electrodes on the medial, lateral, and long heads of the triceps brachii. Amp, amplifier.

The MVC force of isometric elbow extension was measured with a custom-designed dynamometer (S-17008, Takei Scientific Instruments Co., Ltd., Niigata, Japan) with a mounted force transducer (TSA-210, Takei Scientific Instruments Co., Ltd., Niigata, Japan). Each subject sat on a dynamometer and flexed the elbow to a given angle, i.e. 60°, 90° or 120°, with the shoulder and wrist set at 45° abduction and in the neutral positions, respectively, and fixed by a strap (Fig 1). The order of joint angles was randomized for each subject. During the task, the trunk and pelvis were strapped to the dynamometer, with the angle between the trunk and arm set at 45°. The MVC force test was performed at 60°, 90°, and 120° extension of the right arm, and the force signal was recorded at the head of the ulna. The elbow extension torque (Nm) by the exerted force (N) and the forearm length (m) was calculated. The forearm length was defined as the length between the lateral epicondyle of the humerus and the radial styloid. The MVC test consisted of the force-rising phase (1–2 s), the sustained phase (≥ 2 s), and the relaxation phase. Vigorous encouragement was given by the supervisors when the force reached the sustained phase. Subjects performed two MVC trials with ≥ 2 min rest between trials. If the two MVCs differed by ≥ 5% between trials, additional trials were performed to meet the criterion [14, 24].

At ≥ 5 min after each MVC test, the subjects performed isometric force matching tasks at 20%, 40%, 60%, and 80%MVC. For 20% to 60%MVC force levels, subjects performed three trials for each contraction level with at least 2 min of rest between each contraction, and two trials for 80%MVC force levels to avoid muscle fatigue. The order of the contraction levels in the force matching task within a given joint angle was randomized. Subjects were allowed more than 2 min of rest between submaximal trials if requested. The subjects were also allowed to release their experimental arm from the dynamometer to relax or stretch the arm during the experiment. The elbow extension force, elbow joint angle measured by an electrogoniometer (SG150, Biometrics, Ltd. Gwent, UK), and surface EMG signals (see section below) were simultaneously sampled at 2 kHz using an A/D converter (PowerLab 16SP, ADInstruments, Melbourne, Australia), and stored on a personal computer (Mac mini, Apple Inc., Cupertino, CA, USA) using commercial software (LabChart, version 7.2.5, ADInstruments). The produced force and the target line for matching force were shown to the subjects in real-time on a 17-inch computer monitor as visual feedback.

Subjects performed a total of six MVC contractions at three joint angles, and 33 force matching tasks at four submaximal force levels. During the experiment, we repeatedly asked the subjects whether they felt muscle fatigue. The MVC force values at an elbow joint angle of 90° before and immediately after the end of the experiment did not significantly differ (21.4 ± 3.2 Nm and 22.1 ± 2.8 Nm; P = 0.393). Thus, we determined that muscle fatigue was unlikely to have affected the results.

The elbow flexion MVC task was performed to examine the degree of co-contraction during the isometric elbow extension task. The subjects performed two elbow flexion MVC trials with ≥ 2 min rest between trials, in the same way as for the elbow extension task.

### EMG recording

Active electrodes were used to record the surface EMG signals from the TB-Med, TB-Lat, and TB-Long during the isometric elbow extension task. The recording used a single differential electrode sensor (4.1 cm long, 2.0 cm wide, 0.5 cm high) with a 1-cm interelectrode distance, and input impedance of > 10^15^ Ω/0.2 pF, a 90 dB common rejection ratio, and frequency response of 20 ± 5 to 450 ± 50 Hz (DE-2.1, Delsys, Inc., Boston, MA, USA). The sensor pre-amplifier was set at a gain of 10-fold, and the main amplifier units was set at a gain of 100-fold. Thus, the original EMG signal was amplified by 1,000-fold.

**Fig 2:**
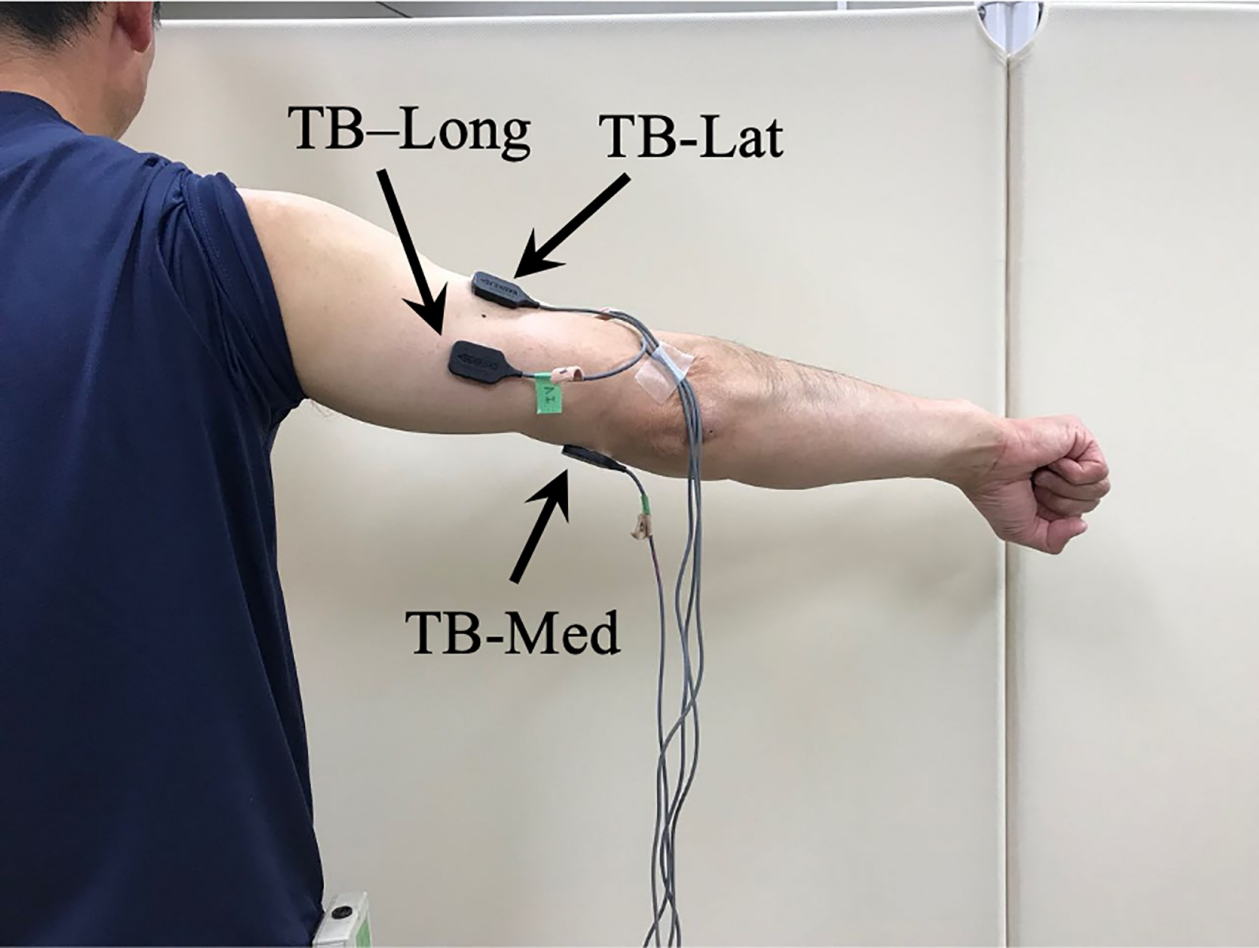
Placement of surface electrodes on the medial, lateral, and long heads of the triceps brachii (TB).

**Fig 3:**
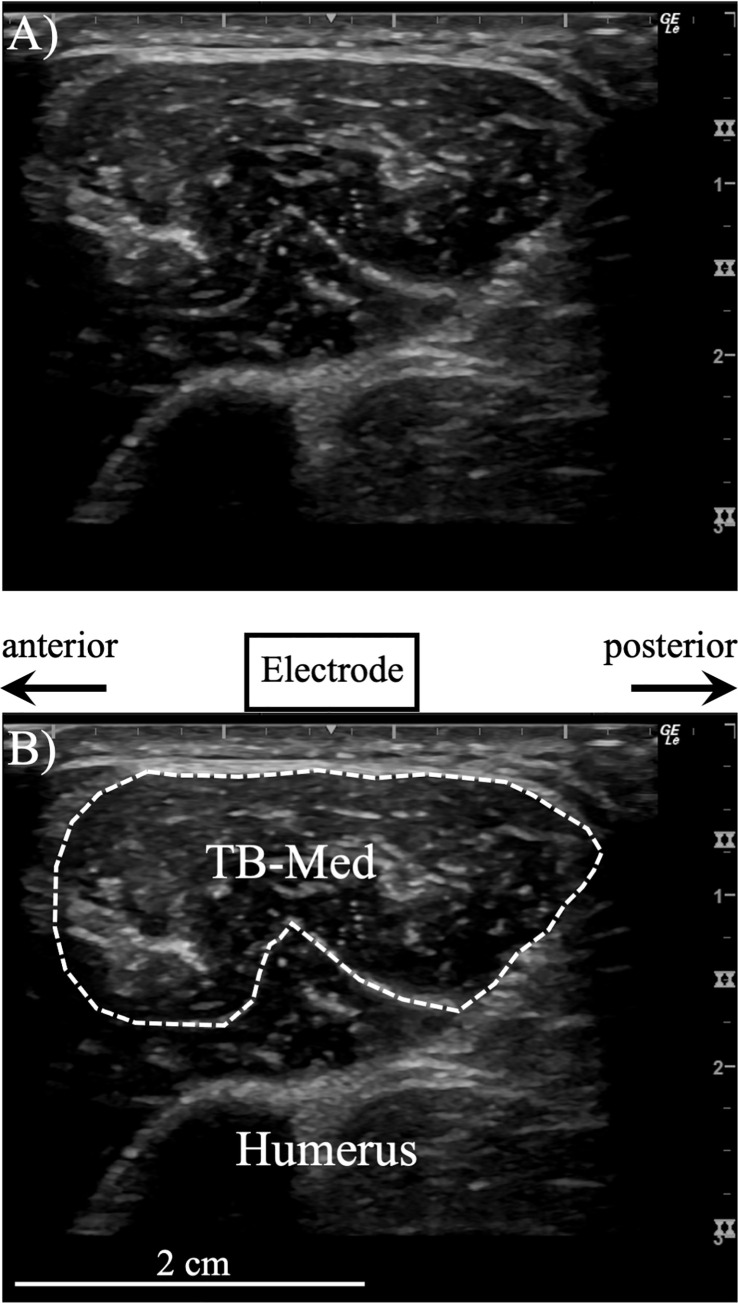
Representative B-mode ultrasound images. A) The original image. B) An image with information regarding the medial head of the triceps brachii (TB-Med) of a male subject. The electrode is 1 cm long and 0.5 cm high, and the long axis length of the TB-Med muscle is 3.1 cm.

The electrodes for the TB-Lat, and TB-Long were placed in accordance with the locations recommended by the Surface electromyography for the Non-Invasive Assessment of Muscles (SENIAM) [25]; the location of the electrode for the TB-Med was also in accordance with previous studies [8, 26–28] (Fig 2). For the TB-Med, ultrasonography was used to determine the superficial region of the muscle that faced the skin surface to minimize cross-talk (Fig 3) [29] and followed the method of previous studies that measured the EMG signal of the TB-Med using a surface EMG electrode [21, 28]. These previous studies used surface electrodes with a 2-cm interelectrode distance and found different EMG signal changes between the TB-Long and TB-Lat with muscle fatigue. This suggests that there is only a small amount of cross-talk from adjacent muscles when using this type of electrode with a 2-cm interelectrode distance. As shown in Fig 3, the best location to place the EMG electrode with a 1-cm interelectrode distance for the TB-Med was determined based on the volume conductance. Our previous studies confirmed that there is negligible cross-talk from adjacent muscles when the width of the target muscle is 3.2 ± 0.4 cm (n = 45) and the interelectrode distance of the surface electrodes is 1 cm [1, 15, 24, 29–33]. The reference electrode was attached to the patella.

### Data analysis

EMG signals were full-wave rectified and the RMS of the EMG signal and MVC force was sampled over 1,000 ms in the same period where the MVC force was seen. The RMS was calculated as previously described [34].

**Fig 4:**
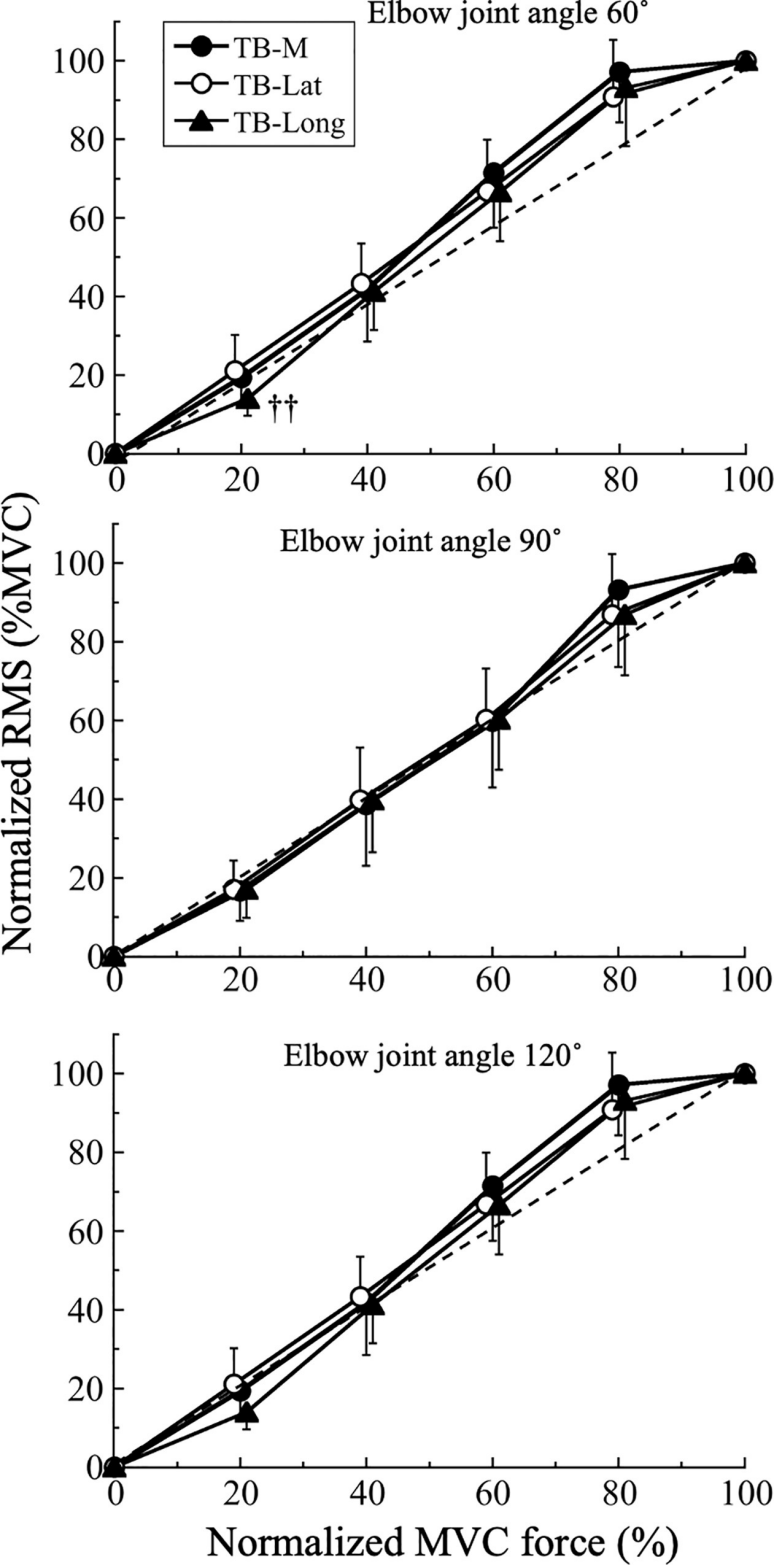
Comparisons of normalized root mean square (RMS) of electromyographic signals and normalized isometric maximum voluntary contraction (MVC) force relationships with respect to the medial (TB-Med), lateral (TB-Lat), and long (TB-Long) heads of the triceps brachii at 60°, 90°, and 120° of extension. ††, P < 0.01 vs TB-Lat; a, P < 0.001 vs other muscles.

The RMS was calculated using EMG signals during the two highest MVC forces, which were averaged to provide a representative value. The RMS values of the TB-Med, TB-Lat, and TB-Long during submaximal force exertion were normalized using the RMS of the MVC during isometric elbow extension for each joint angle for the TB-Med, TB-Lat, and TB-Long. MVC force during submaximal force levels was also expressed as normalized values with respect to MVC force (e.g. Figs 3 and 4).

The normalized RMS and normalized MVC force relationships, i.e. normalized RMS-force relationship, were determined at each elbow joint angle for comparisons between muscles for each joint angle and for comparisons between joint angles for each muscle. The sum of difference was also calculated using the following equation [4, 14, 15, 31].

$$\begin{array}{r} Sum\;of\;difference=\Sigma |A_{i}‐B_{i}|\\ i=20,40,60,80 \end{array}$$

where Ai and Bi are the normalized RMS of the EMG signals and the normalized performed elbow extension force at i% of MVC, respectively. The sum of difference between the performed force and RMS was compared between the three TB muscles to assess the shape or linearity of the normalized RMS to normalized force relationship.

### Statistics

All data are presented as mean ± standard error. The Shapiro-Wilk test was used to examine the normality of data distribution. One-way analysis of variance (ANOVA) was used to compare MVC torques among tested three different elbow joint angles. Two-way (muscle × force level) ANOVA with repeated measurements was used to compare the RMS between muscles at each joint angle and between joint angles at each muscle. Two-way (muscle × angle) ANOVA was used to compare the sum of difference between joint angles for each muscle. When a two-way interaction or main effects were found, the Tukey post-hoc test was used to identify significant differences. The Mauchly sphericity test was applied; when violated, the Greenhouse-Geisser correction factor was used to control for type I errors. The partial eta squared (η^2^) statistic was used to evaluate the effect size for each ANOVA. The effect size was defined as small if η^2^ < 0.01, medium when η^2^ < 0.06, and large when η^2^ < 0.14 [35]. The level of significance was set as P < 0.05. Statistical analyses were performed with SPSS software (version 26.0; IBM, Tokyo, Japan).

## Results

Table 1 shows the exerted force and joint angles during the tasks. MVC torque were 20.2 ± 2.7 Nm, 22.1 ± 2.8 Nm, and 18.6 ± 2.3 Nm at joint angles of 60°, 90° and 120°, respectively. There were no significant differences in MVC torque among the joint angles. Throughout the experiment, the exerted forces were similar to the target forces and the joint angles were kept at the specified angles. This shows that our experiment was strictly controlled.

**Table 1:** Exerted force and joint angle during elbow extension tasks.

				Exerted force (%)									Joint angle (°)					
		60°			90°			120°			60°			90°			120°	
20%	23.9	±	0.3	23.3	±	0.4	24.5	±	0.4	63.6	±	1.0	88.4	±	1.3	119.5	±	1.5
40%	42.5	±	0.5	41.4	±	0.4	42.3	±	0.6	63.6	±	1.1	88.4	±	1.5	119.9	±	1.2
60%	62.6	±	0.6	61.0	±	0.7	62.5	±	0.8	64.1	±	1.5	89.9	±	1.5	120.1	±	1.4
80%	82.5	±	0.9	80.1	±	1.1	82.5	±	0.8	63.9	±	1.9	90.8	±	1.5	120.7	±	1.8
Values are means and SE.																		

Data are presented as mean ± standard error.

Fig 4 shows the relationship between the normalized RMS and normalized MVC force in each muscle for each joint angle. There were significant force effects (60°, F_2,127_ = 984.2, P = 0.001, η^2^ = 0.943; 90°, F_2,146_ = 773.5, P = 0.001, η^2^ = 0.928; 120°, F_2,138_ = 620.2, P = 0.001, η^2^ = 0.913), muscle effects (60°, F_3,60_ = 143.2, P = 0.001, η^2^ = 0.877; 90°, F_3,60_ = 84.8, P = 0.001, η^2^ = 0.773; 120°, F_3,59_ = 72.6, P = 0.001, η^2^ = 0.787), and force-by-muscle interactions (60°, F_2,127_ = 984.2, P = 0.001, η^2^ = 0.818; 90°, F_2,146_ = 68.2, P = 0.001, η^2^ = 0.809; 120°, F_2,138_ = 55.9, P = 0.001, η^2^ = 0.740).

Post-hoc comparison revealed that the RMS of the TB-Long during 20%MVC at 60° was significantly lower than that of the TB-Lat (P = 0.007).

**Fig 5:**
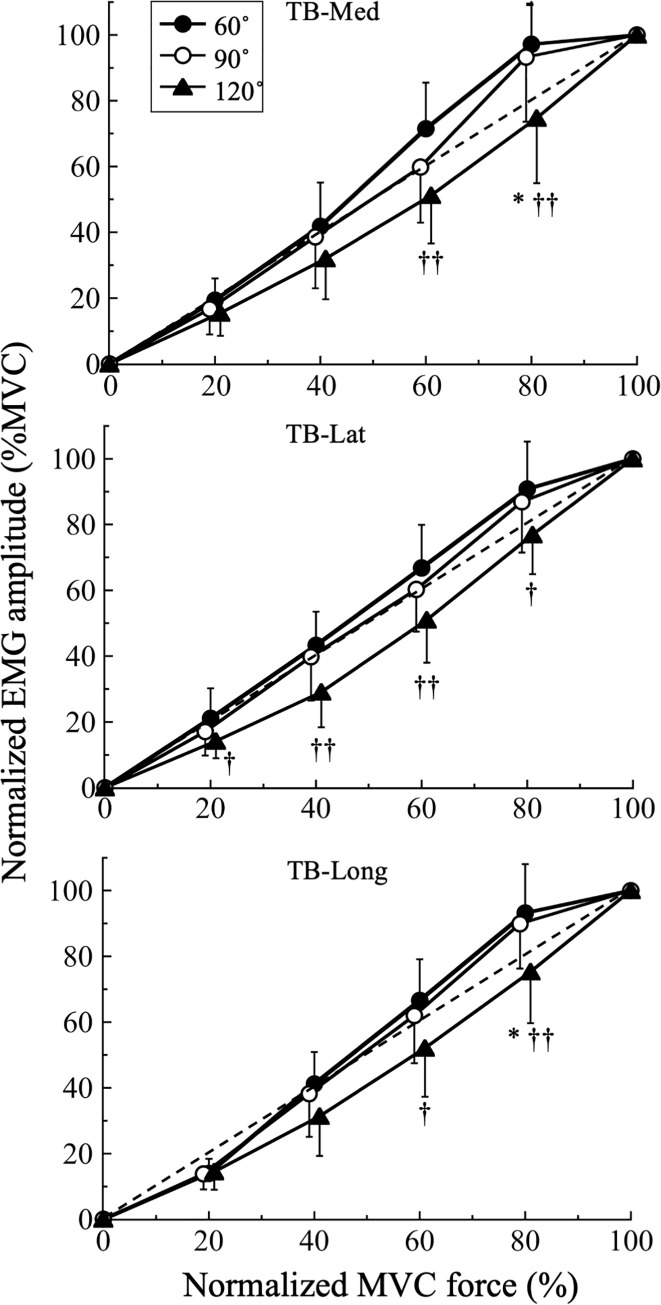
Comparisons of normalized root mean square (RMS) of electromyographic signal and normalized isometric maximum voluntary contraction (MVC) force relationships with respect to joint angles of 60°, 90°, and 120° of extension for the medial (TB-Med), lateral (TB-Lat), and long (TB-Long) heads of the triceps brachii. †, P < 0.05, ††, P < 0.01 vs 60°; *, P < 0.05, **, P < 0.01, vs 90°.

Fig 5 shows the relationship between normalized RMS and normalized MVC force at various joint angles for each muscle. There were significant force effects (TB-Med, F_2,106_ = 621.5, P = 0.001, η^2^ = 0.932; TB-Lat, F_3,111_ = 743.2, P = 0.001, η^2^ = 0.943; TB-Long, F_3,98_ = 948.3, P = 0.001, η^2^ = 0.956), angle effects (TB-Med, F_2,45_ = 5.7, P = 0.006, η^2^ = 0.203; TB-Lat, F_2,45_ = 6.8, P = 0.003, η^2^ = 0.233; TB-Long, F_2,44_ = 5.1, P = 0.010, η^2^ = 0.189). Force-by-angle interactions (TB-Med, F_6,106_ = 5.4, P = 0.001, η^2^ = 0.194; TB-Long, F_6,98_ = 6.0, P = 0.001, η^2^ = 0.213) was also found, except for the TB-Lat (TB-Lat, F_5,111_ = 1.5, P = 0.196, η^2^ = 0.063).

Post-hoc comparison revealed that the RMS of the TB-Med during 60%MVC (P = 0.001) and 80%MVC (P = 0.002) at 120° were significantly lower than the values at 60°. The RMS during 80%MVC at 120° was also significantly lower than that at 90° (P = 0.013). The RMS of the TB-Lat during all force levels at 120° were significantly lower than the values at 60° (20%MVC, P = 0.030; 40%MVC, P = 0.003; 60%MVC, P = 0.004; 80%MVC, P = 0.019). The RMS of the TB-Long during 60%MVC (P = 0.014) and 80%MVC (P = 0.004) at 120° were significantly lower than the values at 60°. The RMS during 80%MVC at 120° was also significantly lower than that at 90° (P = 0.022).

**Fig 6:**
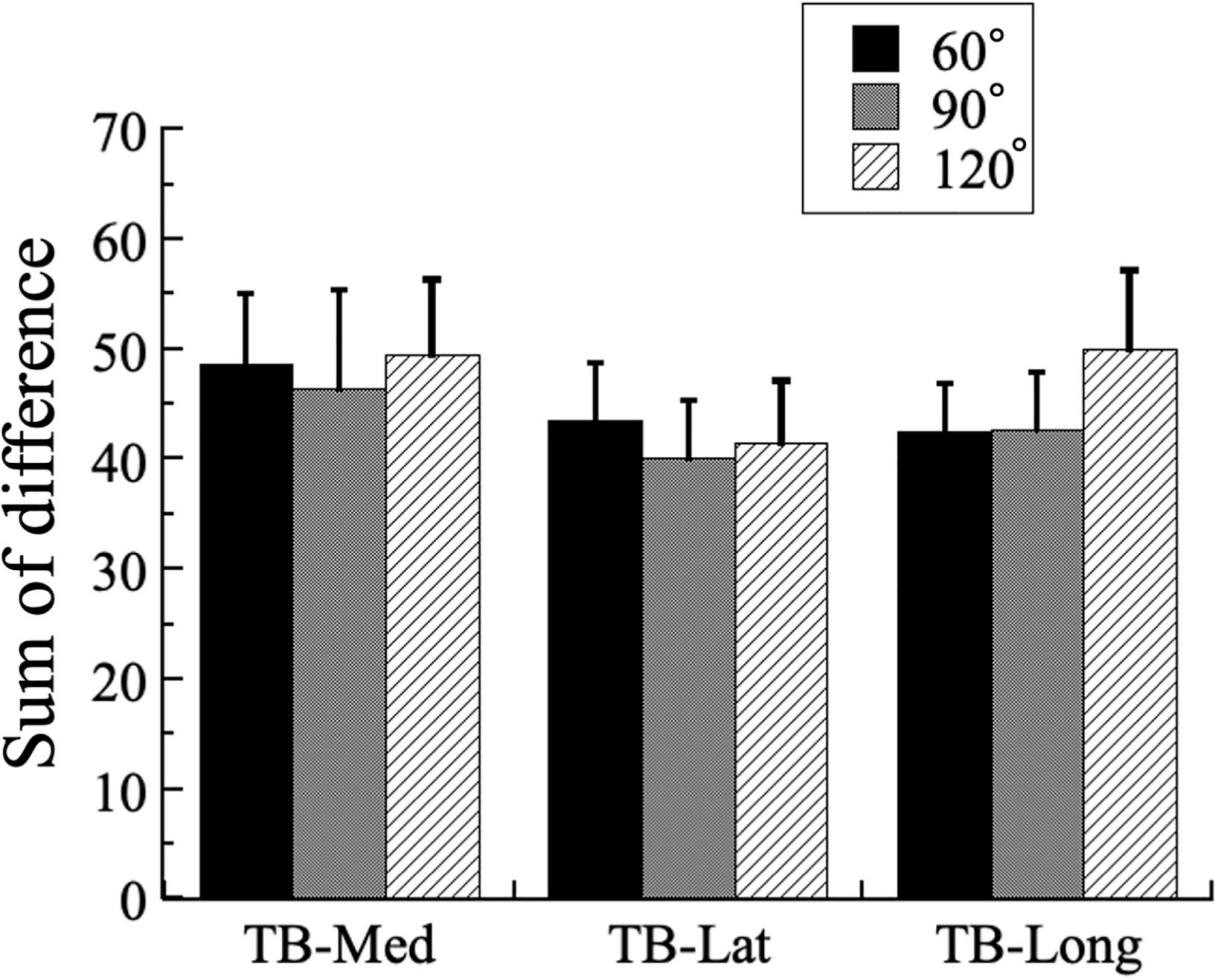
Sum of difference for the medial (TB-Med), lateral (TB-Lat), and long (TB-Long) heads of the triceps brachii between normalized root mean square (RMS) of electromyographic signal and normalized isometric maximum voluntary contraction force relationships at 60°, 90°, and 120° of extension.

The sum of difference results are shown in Fig 6. There were no muscle effects (F_2,144_ = 0.791, P = 0.456, η^2^ = 0.012), angle effects (F_2,144_ = 0.301, P = 0.741, η^2^ = 0.004), or muscle-by-angle interactions (F_4,144_ = 0.155, P = 0.960, η^2^ = 0.005).

## Discussion

The present study aimed to assess the normalized RMS-force relationships of the TB-Med, TB-Lat, and TB-Long during isometric elbow extensions of 60°, 90°, and 120°. The main finding was that the normalized RMS-force relationships were similar in the three muscles at each joint angle; however, the normalized RMS-force relationship at 120° was significantly lower than that at 60° and 90° at a few force levels in each muscle. For each muscle, the sum of difference did not significantly differ between the three joint angles.

### Normalized RMS-force relationships in the three muscles

There were no significant differences between the normalized RMS-force relationships of the TB-Med, TB-Lat, and TB-Long at each joint angle. This result was contrary to our hypothesis that the TB-Med would shift downward with elbow extension based on our previous study [14].

We found that the RMS of each muscle increased linearly with increasing force (Figs 4 and 5), suggesting that force control was finely modulated by the recruitment and/or firing rates of the MUs of each muscle. This result also suggests that there may be no difference in such force control strategies among the three TB muscles. However, it is reported that the normalized RMS-force relationship is complex and dependent on the tested muscle. Lawrence and De Luca (1983) found that the normalized RMS-force relationship was quasilinear, i.e. almost linear, in the first dorsal interosseous muscle, but nonlinear in the biceps brachii and deltoid muscles. Another study that assessed the normalized RMS-force relationship of the four individual quadriceps muscles found that the normalized RMS-force relationship of the vastus intermedius gradually shifted downward with knee extension compared with the other synergistic muscles [14]. The downward shift of the vastus intermedius was also confirmed using an analysis of the sum of difference, which means that the degree of absolute difference from the identical line became larger with further extension of the knee. This result conflicts with the present findings in which the sum of difference did not significantly differ between the TB muscles. The reasons for the conflicting results may be physiological, biomechanical, and task-dependent. There seems to be no difference in muscle fiber composition between the TB (32.5% type I fibers) and vastus lateralis (37.8% type I fibers). However, the TB is a non-antigravity muscle, while the quadriceps femoris is an antigravity muscle. Task-dependent effects, including experimental settings, may have a significant impact on muscle function and MU activation properties.

The muscle contraction force is mainly controlled by recruitment, firing rate, and/or synchronization of MUs [36–38]. A previous study demonstrated that the force control strategy is mainly modulated by recruitment of MUs in large muscles such as the biceps brachii, at least until it reaches 90% of its contractile force range [39]. However, no further MU recruitment is observed above 50%MVC in small muscles such as the adductor pollicis [39]. A previous study showed no difference in MU recruitment threshold between the TB-Lat and TB-Long during isometric elbow extension at up to 75%MVC [13]. In the present study, although we did not measure MU recruitment thresholds, we did not find a significant difference in the normalized RMS-force relationships between the TB-Med, TB-Lat, or TB-Long at each force level. Furthermore, the TB-Lat and TB-Long have similar architecture and functional properties [13, 40, 41]. Similarly, another study demonstrated significant relationships between muscle force and an index of MU size from 5–100% of MVC in 208 MUs in the vastus medialis (i.e. a larger muscle) using a needle electrode [42]. The index of MU size explained 67% of muscle force variance (i.e. r^2^ = 0.670) based on a simple regression analysis. Furthermore, when combining the mean firing rates of MUs with an index of MUs size, there is no further increase in the correlation coefficient (r^2^ = 0.706, P < 0.0001), and the MU firing rate alone explains only 38% of muscle force variance of the quadriceps femoris during isometric contractions (r^2^ = 0.384) [42]. These previous studies clearly show that MU recruitment is the major determinant of muscle force from smaller to greater levels of force production in larger muscles. The TB is categorized as a large muscle [39], and so MU recruitment is expected to play a crucial role in controlling force production. Thus, the normalized RMS-force relationship of the synergistic TB muscles assessed in the present study is likely to be linear at all joint angles, suggesting that the recruitment and firing rate of MUs functioned smoothly during the task.

The force-sarcomere length relationship also affects the EMG-force relationship. Previous study of the force-generating potential of sarcomeres of the TB-Long and TB-Lat at the plateau phase of the normalized force-length relationships from 60° to 150° suggests that the sarcomere length-associated force generating capacity is able to be excluded as an influential factor to affect the normalized RMS-force relationship [43]. Because, the force generating capacity of the sarcomeres is considered to be similar to the range of motion assessed in the present study. Although no data were obtained regarding the force-sarcomere length relationship for the TB-Med, the TB-Med is likely to have a similar force-sarcomere length relationship as the TB-Long and TB-Lat. This may be one of the reasons for the linearity between the normalized RMS-force relationship in the present study.

### RMS-force relationship at various elbow joint angles

Overall, the normalized RMS at 120° was significantly lower than that at 60° or 90°, but not for 100%MVC force, in the TB-Med, TB-Lat, and TB-Long. However, there was no muscle-specific difference in the normalized RMS-force relationship across joint angles, which conflicts with our hypothesis and previous findings for the quadriceps [14].

The RMS of the TB-Lat at 25%, 50%, 75%, and 100%MVC at an elbow joint angle of 120° have previously been reported [17]. When the RMS data reported in this previous study were converted to enable comparison with the present data, those values were approximately 28%, 50%, and 75% at 25%MVC, 50%MVC, and 75%MVC, respectively. This suggests that the relative force exertion levels were closely matched with the RMS, which conflicts with the findings of the current study. This difference may be caused by interstudy variations in posture during the experiment. The present subjects sat on a dynamometer with their elbow flexed at a given angle and the wrist in the neutral position (Fig 1). In contrast, the subjects in the previous study sat in a chair with the forearm supinated and the shoulder restrained by straps [17]. Interestingly, Doheny et al. [8] used an experimental posture that was similar to that used in the present study and found a significant effect on the normalized RMS-force relationship at 150° and 170°, suggesting that a shorter muscle length affects the relationship.

Some previous studies focused on the MUs of the TB-Lat. For example, Le Bozec and Maton [23] evaluated the integrated EMG-force and MU firing frequency-force relationships up to 30%MVC at an elbow joint angle of 90° and demonstrated that the integrated EMG increased continuously with increased force, so that the mean MU firing rates rose steadily and continuously with torque. Furthermore, the minimal firing rates were 8–16 Hz, while the maximal firing rates were 14–16 Hz at torque values corresponding to 30%MVC. Del Valle and Thomas [17] also evaluated the MU firing rates of the TB-Lat during isometric contractions at different elbow joint angles. The average MU firing rates increased from 15.6 ± 0.3 to 22.7 ± 0.7 Hz from 25%MVC to 100%MVC during elbow extension while exerting force at five joint angles. Importantly, they also suggested that newly activated MUs can be recruited up to at least 75%MVC, because many of the MUs fired at rates commonly found in 100%MVC. The main factors contributing to the downward shift of the normalized RMS-force relationship at 120° in all three muscles in the present study could not be identified, but may have been affected by experimental posture [17].

Saito and Akima [14] reported that the normalized RMS-force relationship shifts downward at some force levels in all four individual muscles of the quadriceps femoris at the shortest muscle length (extended knee joint angle). This result is similar to the current study in that shorter muscle length induced a downward shift in the normalized RMS-force relationship. Vander Linden et al. [18] examined the effect of muscle length on MU discharge characteristics of the tibialis anterior muscle. They showed a significant relationship between the MU discharge rate and dorsiflexion torque; during isometric contractions, the MU discharge rate was greater per unit torque at the shortened muscle length versus the neutral length or lengthened muscle. This suggest that the MU discharge is less efficient at shorter muscle lengths. This result clearly demonstrates that changes in muscle length affect the MU firing characteristics.

### Sum of difference

The sum of difference is a useful indicator of the difference in RMS from the identical line, which enables the quantitative analysis of the shape of the normalized RMS-force relationship. The sum of difference did not significantly differ in any muscle assessed in the present study. In contrast, previous studies that assessed the normalized RMS-force relationship revealed a significant difference in the sum of difference between the vastus intermedius and rectus femoris at a knee joint angle of 90° [31]. Also, the normalized RMS-force relationship between the vastus intermedius and vastus lateralis, vastus medialis, or rectus femoris at a shorter muscle length compared with that at 90° or larger [14]. Furthermore, there were reportedly no significant differences between any assessed muscles during the performance of isotonic knee extension tasks in the concentric or eccentric phases with loads from 20% to 100% during one repetition [15]. These conflicting results may be related to the physical characteristics of the subjects, target muscles, contraction type, or joint angles (i.e. sarcomere length-force relationship).

## Conclusions

The normalized RMS-force relationship of three synergistic TB muscles during isometric elbow extension at three joint angles did not significantly differ between muscles for each joint angle. When compared between joint angles, the normalized RMS-force relationship shifted downward primarily at greater force levels at 120° in each muscle. The sum of difference did not significantly differ between the three assessed angles for each muscle. These results suggest that each TB synergist is not likely to have a specific function at greater or smaller force levels; however, the RMS decreased in tandem with the shortening muscle length. These results suggest that a relatively greater RMS was required when the elbow joint angle was extended. However, RMS and relative force levels were matched in all three TB synergists when the elbow joint angle was at 90° or a more extended position. In summary, the normalized RMS-force relationships of all three individual muscles of the TB shifts downward at 60%MVC and 80%MVC when extended elbow joint angles. The results of this study may contribute to the establishment of future neuromusculoskeletal modelling for estimating muscle force from EMG. As the TB is the main muscle used when self-propelling a wheelchair, the findings of the present study also provide basic data not only for members of the general public who use wheelchairs, but also for paralympians.

## Supporting information

S1 Data We included surface EMG data with respect to different angles and sum of difference data. (XLSX)

## References

[1] Watanabe K, Akima H. Effect of knee joint angle on neuromuscular activation of the vastus intermedius muscle during isometric contraction. Scand J Med Sci Sports. 2011;21:e412–e20. doi: 10.1111/j.1600-0838.2011.01347.x 21672026

[2] De Luca CJ. The use of surface electromyography in biomechanics. J Appl Biomech. 1997;13:135–63. doi: 10.1123/jab.13.2.135

[3] Lawrence JH, DeLuca CJ. Myoelectric signal versus force relationship in different human muscles. J Appl Physiol. 1983;54:1653–9. doi: 10.1152/jappl.1983.54.6.1653 6874489

[4] Alkner BA, Tesch PA, Berg HE. Quadriceps EMG/force relationship in knee extension and leg press. Med Sci Sports Exerc. 2000;32:459–63. doi: 10.1097/00005768-200002000-00030 10694132

[5] Komi PV, Viitasalo JHT. Signal characteristics of EMG at different levels of muscle tension. Acta Physiol Scand. 1976;96:267–76. doi: 10.1111/j.1748-1716.1976.tb10195.x 1258672

[6] Pincivero D, Coelho AJ. Activation linearity and parallelism of the superficial quadriceps across the isometric intensity spectrum. Muscle Nerve. 2000;23:393–8. doi: 10.1002/(sici)1097-4598(200003)23:3&lt;393::aid-mus11&gt;3.0.co;2-p 10679716

[7] Pincivero DM, Coelho AJ, Campy RM, Salfetnikov Y, Suter E. Knee extensor torque and quadriceps femoris EMG during perceptually-guided isometric contractions. J Electromyogr Kinesiol. 2003;13(2):159–67. doi: 10.1016/s1050-6411(02)00096-2 12586521

[8] Doheny EP, Lowery MM, Fitzpatrick DP, O’Malley MJ. Effect of elbow joint angle on force-EMG relationships in human elbow flexor and extensor muscles. J Electromyogr Kinesiol. 2008;18(5):760–70. Epub 2007/05/15. doi: 10.1016/j.jelekin.2007.03.006 . 17499516

[9] Praagman M, Veeger HEJ, Chadwick EKJ, Colier WNJM, van der Helm FCT. Muscle oxygen consumption, determined by NIRS, in relation to external force and EMG. J Biomech. 2003;36(7):905–12. doi: 10.1016/s0021-9290(03)00081-2 12757798

[10] Woods JJ, Bigland-Ritchie B. Linear and non-linear surface EMG/force relationships in human muscles. An anatomical/functional argument for the existence of both. Am J Phys Med. 1983;62:287–99. PubMed Central PMCID: PMC6650674. 6650674

[11] De Luca CJ, LeFever RS, McCue MP, Xenakis AP. Behaviour of human motor units in different muscles during linearly varying contractions. J Physiol (London). 1982;329:113–28. doi: 10.1113/jphysiol.1982.sp014293 PubMed Central PMCID: PMC1224770. 7143246 PMC1224770

[12] Harridge SDR, White MJ. Muscle activation and the isokinetic torque-velocity relationship of the human triceps surae. Eur J Appl Physiol. 1993;67:218–21. doi: 10.1007/BF00864218 8223533

[13] Harwood B, Dalton BH, Power GA, Rice CL. Motor unit properties from three synergistic muscles during ramp isometric elbow extensions. Exp Brain Res. 2013;231(4):501–10. Epub 2013/10/02. doi: 10.1007/s00221-013-3714-y . 24081681

[14] Saito A, Akima H. Knee joint angle affects EMG-force relationship in the vastus intermedius muscle. J Electromyogr Kinesiol. 2013;23:1406–12. doi: 10.1016/j.jelekin.2013.08.009 24075525

[15] Akima H, Saito A. Inverse activation between the deeper vastus intermedius and superficial muscles in quadriceps during dynamic knee extensions. Muscle Nerve. 2013;47:682–90. doi: 10.1002/mus.23647 23504547

[16] Miles TS, Nordstrom MA, Türker KS. Length-related changes in activation threshold and wave form of motor units in human masseter muscle. J Physiol (London). 1986;370:457–65. doi: 10.1113/jphysiol.1986.sp015944 PubMed Central PMCID: PMC1192690. 3958983 PMC1192690

[17] Del Valle A, Thomas CK. Motor unit firing rates during isometric voluntary contractions performed at different muscle lengths. Can J Physiol Pharmacol. 2004;82(8–9):769–76. Epub 2004/11/04. doi: 10.1139/y04-084 . 15523534

[18] Vander Linden DW, Kukulka CG, Soderberg GL. The effect of muscle length on motor unit discharge characteristics in human tibialis anterior muscle. Exp Brain Res. 1991;84:210–8. doi: 10.1007/BF00231776 1855559

[19] Kawakami Y, Abe T, Fukunaga T. Muscle-fiber pennation angles are greater in hypertrophied than in normal muscles. J Appl Physiol. 1993;74:2740–4. doi: 10.1152/jappl.1993.74.6.2740 8365975

[20] Kawakami Y, Abe T, Kuno S, Fukunaga T. Training-induced changes in muscle architecture and specific tension. Eur J Appl Physiol. 1995;72:37–43. doi: 10.1007/BF00964112 8789568

[21] Hussain J, Sundaraj K, Subramaniam ID, Lam CK. Muscle fatigue in the three heads of triceps brachii during intensity and speed variations of triceps push-down exercise. Front Physiol. 2020;11:112. Epub 2020/03/11. doi: 10.3389/fphys.2020.00112 ; PubMed Central PMCID: PMC7047337. 32153422 PMC7047337

[22] Wade MD, McDowell AR, Ziermann JM. Innervation of the Long Head of the Triceps Brachii in Humans-A Fresh Look. Anat Rec (Hoboken). 2018;301(3):473–83. Epub 2018/02/09. doi: 10.1002/ar.23741 . 29418118

[23] Le Bozec S, Maton B. Differences between motor unit firing rate, twitch characteristics and fibre type composition in an agonistic muscle group in man. Eur J Appl Physiol. 1987;56:350–5. doi: 10.1007/BF00690904 3569244

[24] Akima H, Ando R. Oxygenation and neuromuscular activation of the quadriceps femoris including vastus intermedius during a fatiguing contraction. Clin Physiol Func Imaging. 2017;37:750–8. doi: 10.1111/cpf.12370 27194371

[25] Hermens HJ, Freriks B. Surface electromyography for the non-invasive sssessment of muscles Enschede, Netherlands1999. Available from: http://www.seniam.org/.

[26] Ali MA, Sundaraj K, Ahmad RB, Ahamed NU, Islam MA, Sundaraj S. Evaluation of repetitive isometric contractions on the heads of triceps brachii muscle during grip force exercise. Technol Health Care. 2014;22(4):617–25. Epub 2014/07/06. doi: 10.3233/THC-140833 . 24990168

[27] Ali MA, Sundaraj K, Badlishah Ahmad R, Ahamed NU, Islam A, Sundaraj S. Muscle fatigue in the three heads of the triceps brachii during a controlled forceful hand grip task with full elbow extension using surface electromyography. J Hum Kinet. 2015;46:69–76. Epub 2015/08/05. doi: 10.1515/hukin-2015-0035 ; PubMed Central PMCID: PMC4519223. 26240650 PMC4519223

[28] Hussain J, Sundaraj K, Subramaniam ID. Cognitive stress changes the attributes of the three heads of the triceps brachii during muscle fatigue. PLoS One. 2020;15(1):e0228089. Epub 2020/01/31. doi: 10.1371/journal.pone.0228089 ; PubMed Central PMCID: PMC6992167. 31999750 PMC6992167

[29] Watanabe K, Akima H. Cross-talk from adjacent muscle has a negligible effect on surface electromyographic activity of vastus intermedius muscle during isometric contraction. J Electromyogr Kinesiol. 2009;19:e280–e9. doi: 10.1016/j.jelekin.2008.06.002 18653357

[30] Saito A, Watanabe K, Akima H. The highest antagonistic coactivation of the vastus intermedius muscle among quadriceps femoris muscles during isometric knee flexion. J Electromyogr Kinesiol. 2013;23:831–7. doi: 10.1016/j.jelekin.2013.02.005 23489717

[31] Watanabe K, Akima H. Normalized EMG to normalized torque relationship of vastus intermedius muscle during isometric knee extension. Eur J Appl Physiol. 2009;106:815–25. doi: 10.1007/s00421-009-1086-6 19404670

[32] Watanabe K, Akima H. Validity of surface electromyography for vastus intermedius muscle assessed by needle electromyography. J Neurosci Methods. 2011;198:332–5. doi: 10.1016/j.jneumeth.2011.03.014 21463655

[33] Watanabe K, Katayama K, Ishida K, Akima H. Electromyographic analysis of hip adductor muscles during incremental fatiguing pedaling exercise. Eur J Appl Physiol. 2009;106:815–25. doi: 10.1007/s00421-009-1086-6 19466446

[34] Basmajian JV, DeLuca CJ. Muscles Alive: Their functions revealed by electromyography. 5th ed. Baltimore, MD: Williams & Wilkins; 1985. 1–561 p.

[35] Stevens JP. Intermediate Statistics: A Modern Approach. 3rd ed. New York, NY: Lawrence Erlbaum Associates; Taylor & Francis Group; 2007. 460 p.

[36] Farina D, Merletti R., Enoka R. The extraction of neural strategies from the surface EMG. J Appl Physiol. 2004;96:1486–95. doi: 10.1152/japplphysiol.01070.2003 15016793

[37] Hägg GM. Interpretation of EMG spectral alterations and alteration indexes at sustained contraction. J Appl Physiol. 1992;73:1211–7. doi: 10.1152/jappl.1992.73.4.1211 1447061

[38] Moritani T, Muro M, Nagata A. Intramuscular and surface electromyogram changes during muscle fatigue. J Appl Physiol. 1986;60:1179–85. doi: 10.1152/jappl.1986.60.4.1179 3700300

[39] Kukulka CG, Clamann HP. Comparison of the recruitment and discharge properties of motor units in human brachial biceps and adductor pollicis during isometric contractions. Brain Res. 1981;219:45–55. doi: 10.1016/0006-8993(81)90266-3 7260629

[40] Srinivasan RC, Lungren MP, Langenderfer JE, Hughes RE. Fiber type composition and maximum shortening velocity of muscles crossing the human shoulder. Clin Anat. 2007;20(2):144–9. Epub 2006/06/24. doi: 10.1002/ca.20349 . 16795030

[41] Terzis G, Georgiadis G, Vassiliadou E, Manta P. Relationship between shot put performance and triceps brachii fiber type composition and power production. Eur J Appl Physiol. 2003;90(1–2):10–5. Epub 2003/05/28. doi: 10.1007/s00421-003-0847-x . 12768426

[42] Conwit RA, Stashuk D, Tracy B, McHugh M, Brown WF, Metter EJ. The relationship of motor unit size, firing rate and force. Clin Neurophysiol. 1999;110:1270–5. doi: 10.1016/s1388-2457(99)00054-1 10423192

[43] Murray WM, Buchanan TS, Delp SL. The isometric functional capacity of muscles that cross the elbow. J Biomech. 2000;33:943–52. doi: 10.1016/s0021-9290(00)00051-8 10828324

